# Construction of DGLA producing cell factory by genetic modification of *Mucor circinelloides*

**DOI:** 10.1186/s12934-019-1110-4

**Published:** 2019-04-03

**Authors:** Md. Ahsanul Kabir Khan, Junhuan Yang, Syed Ammar Hussain, Huaiyuan Zhang, Li Liang, Victoriano Garre, Yuanda Song

**Affiliations:** 1 Colin Ratledge Center for Microbial Lipids, School of Agricultural Engineering and Food Science, Shandong University of Technology, Zibo, 255000 Shandong People’s Republic of China; 2 Departmento de Genética y Microbiología (Unidad Asociada al Instituto de Química Física Rocasolano, Consejo Superior de Investigaciones Científicas), Facultad de Biología, Universidad de Murcia, 30100 Murcia, Spain; 3 State Key Laboratory of Food Science and Technology, School of Food Science and Technology, Jiangnan University, Wuxi, 21412 Jiangsu China

**Keywords:** Delta-6 elongase, DGLA production, Homologous overexpression, *Mucor circinelloides*

## Abstract

**Background:**

Dihomo-gamma linolenic acid (DGLA, 20:3, n-6) is the elongated product of Gamma linolenic acid (GLA, 18:3, n-6) catalyzed by the enzyme delta-6 elongase (D6E) or gamma linolenic acid elongase (GLELO). Construction of engineered oleaginous microbes have been attracting significant interest to produce DGLA because of its nutritional value and medicinal applications. *Mucor circinelloides* is a GLA producing filamentous fungus which can be a useful tool to produce DGLA. We have, therefore, overexpressed the *D6E (GLELO)* gene in this fungus to construct DGLA producing cell factory.

**Result:**

To produce DGLA in *M. circinelloides*, homologous overexpression of *D6E (GLELO)* gene was analyzed. When the gene was overexpressed in *M. circinelloides* CBS277.49, up to 5.72% DGLA was produced in this strain.

**Conclusion:**

To our knowledge, this is the first report describing the overexpression of *D6E (GLELO)* gene in *M. circinelloides* to construct DGLA producing cell factory. A new scope for further research has been established by this work for improved production of DGLA in this fungus, specifically in its high lipid-producing strain, WJ11.

**Electronic supplementary material:**

The online version of this article (10.1186/s12934-019-1110-4) contains supplementary material, which is available to authorized users.

## Introduction

Polyunsaturated fatty acids (PUFAs) are attracting significant interest because they have important roles in human health and nutrition [1]. Mammals, including humans, can synthesize saturated fatty acids (SAFA) and mono-unsaturated fatty acids (MUFA) but cannot synthesize de novo omega-6 or omega-3 polyunsaturated fatty acids (PUFA) which are linoleic acid (LA, 18:2, n-6) and α-linolenic acid (ALA, 18:3, n-3) respectively. Therefore, these PUFA are essential fatty acids (EFAs) for human health and must be obtained from the diet [1, 2]. LA and ALA are metabolised to Docosapentaenoic acid (DPA, 22:5, n-6) and Docosahexaenoic acid (DHA, 22:5, n-3) respectively in a series of reactions catalyzed by the same sets of enzymes [3]. But several diseases such as obesity, hypertension, diabetes mellitus, coronary heart disease, schizophrenia, Alzheimer’s disease, atherosclerosis, and cancer can alter the metabolism of essential fatty acids (EFAs). This indicated that EFAs and their derivatives have various biological activities and appear to be involved in a number of physiological and pathological reactions [4].

A C_20_ PUFA with three double bonds named dihomo-gamma linolenic acid (DGLA, 20:3, n-6) is the elongated product of Gamma linolenic acid (GLA, 18:3, n-6) catalyzed by the enzyme Δ6 elongase [1]. Recently DGLA has attracted significant biological interest becasue it undergoes oxidative metabolism by cyclooxygenases and lipoxygenases to produce anti-inflammatory eicosanoids (prostaglandins of series 1 and leukotrienes of series 3) [5]. DGLA can also be converted to arachidonic acid (AA, 20:4, n-6) by the action of the enzyme delta-5 desaturase. AA forms the precursor of 2 series of prostaglandins, thromboxanes and the 4 series of leukotrienes [6].

Some fungi, microalgae and bacteria can accumulate lipid more than 20% of their cell dry weight (CDW) hence they are called oleaginous microorganisms [7]. *Mucor circinelloides* has been extensively investigated for GLA production since the 1980s [8–10]. Among oleaginous filamentous fungi, it has been considered as an important model organism for lipid accumulation studies due to its ability to produce an oil rich in GLA that may have special effects for the treatment of premenstrual tension, atopic dermatitis and some other diseases and also due to the availability of genome data and genetic tools [7].

Because of nutritional value and medicinal applications many groups are constructing engineered DGLA producing microbes such as recombinant *Aspergillus oryzae*, Saccharomyces cerevisiae and *Mortierella alpina* but all of these strains have some limitations such as lower DGLA producing and problems to separate DGLA from other fatty acids [1, 11, 12]. *M. alpina* can accumulate lipids up to 50% of its dry weight with high amount of arachidonic acid (AA, 20:4, n-6) [13–16]. Some of its essential genes for lipid synthesis have been cloned and partially characterized, and several biochemical reactions have been studied in detail [17]. Delta 6-elongase enzyme is responsible for elongation of GLA (18:3 n-6) to dihomo-gamma-linolenic acid (20:3 n-6), hence the gene is known as *gamma linolenic acid elongase (GLELO)* gene [18]. *M. circinelloides* is a GLA producing filamentous fungus which can be a useful tool to produce DGLA. In this experiment we have cloned *D6E (GLELO)* gene from *M. alpina* and attempted to construct a DGLA producing cell factory by genetic recombination in *M. circinelloides*.

## Results

### Generation of *D6E*-Overexpressing strains of *M. circinelloides* by genetic engineering

**Fig. 1:**
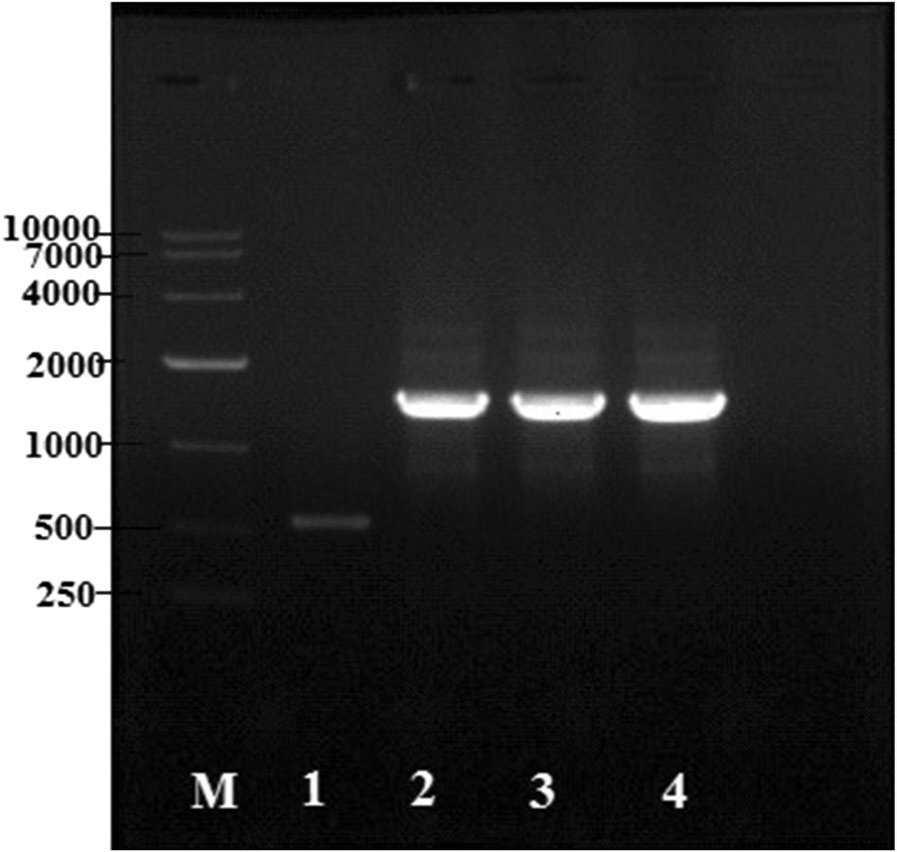
PCR amplification of genome of control and recombinant strains with the primers 1552-F/R. Lane 1 representing the control strain Mc-1552 and 2, 3, 4 showing the presence of *D6E* gene in recombinant strains Mc-D6E, Mc-D6E−1 and Mc-D6E− 2 respectively

Based on the genomic data of *Mortierella alpina*, we found the gene encoding for D6E (GLELO) (Genebank accession number AF206662) which is 957 bp long. To overexpress the target gene, the plasmid pMAT1552-gene that contains the target gene coding region under the control of the strong zrt1 promoter of *M. circinelloides* [8] was generated (Fig. 1). The target gene-overexpressing plasmids, pMAT1552-GLELO and the empty plasmids pMAT1552 was transformed into the uridine auxotrophic strain, pleu-MU402 and selection of the colonies were carried out as described by Rodr´ıguez-Fr´ometa [19].

Integration of the target genes into the genome of overexpression transformants, were confirmed by PCR analysis. Amplification was carried out using a primer pair 1552-F/R (Additional file 1: Table S1) that amplified the target gene and 557 bp sequences of the plasmid pMAT1552. The PCR product fragments for each transformants was 1514 bp as expected, in these corresponding transformants genome, whereas 557 bp fragments was amplified in the control strain Mc-1552 (Fig. 1). PCR amplification results confirmed that the target genes had been integrated into the genome of the fungus in these transformants. For overexpression strain, three transformants were selected, named Mc-D6E, Mc-D6E−1, Mc-D6E−2 and the control transformant that was named as Mc-1552. Additional screening was carried out (data was not mentioned) and only one strain (Mc-D6E) which produced maximum amount of lipid and DGLA, was selected for further experiments.

### Cell growth and lipid accumulation in recombinant *M. circinelloides*

**Fig. 2:**
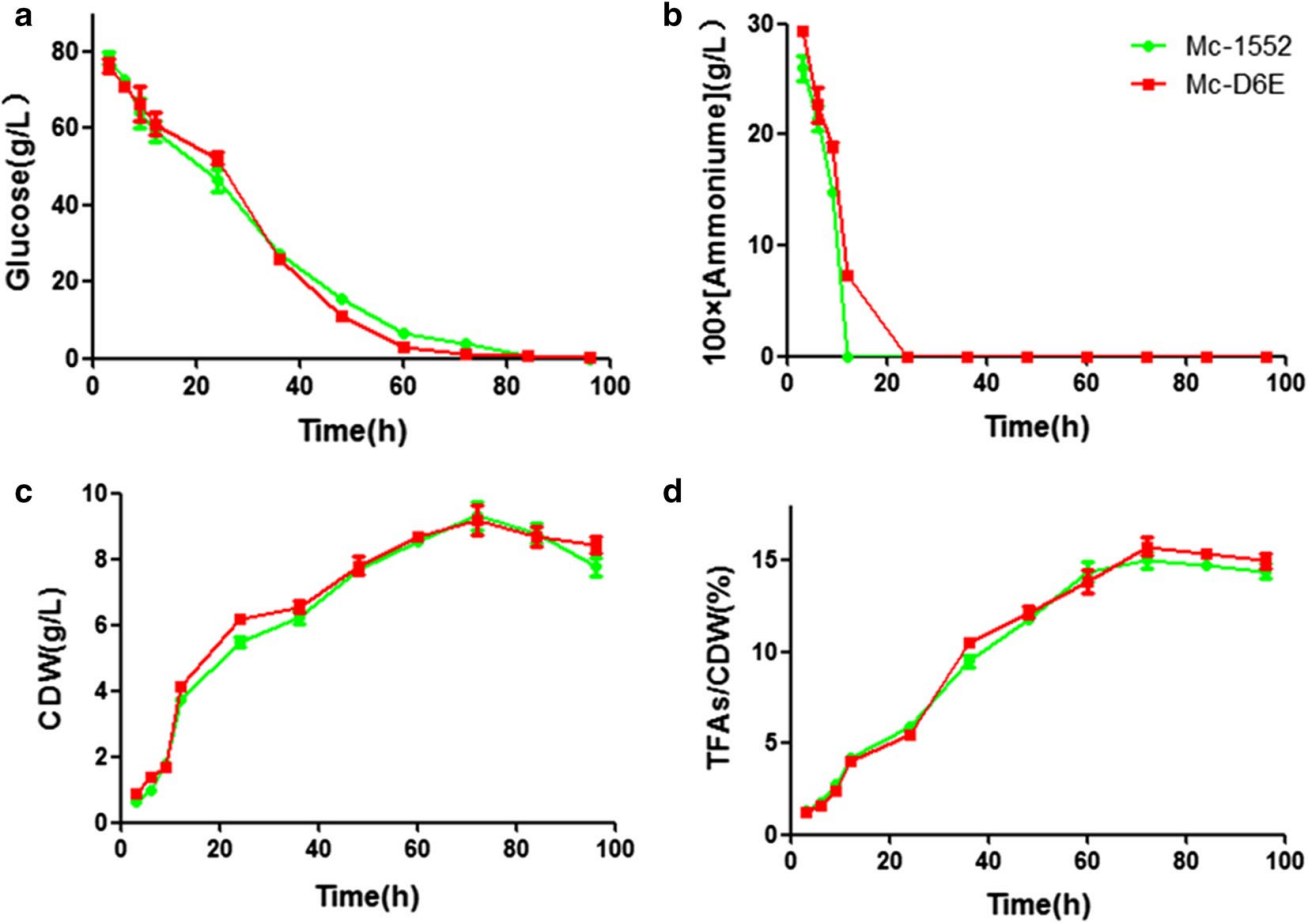
Cell growth and lipid accumulation of *D6E* overexpressing strains. Recombinant Mc-D6E and control strain Mc-1552 cultures were grown in 1.5 L modified K & R medium and **a** glucose concentration, **b** ammonium concentration, **c** cell dry weight (CDW), and **d** Lipid content were measured. Samples from the fermenter were taken at the indicated times. The values were mean of three biological replicates. Error bars represent the standard error of the mean

The concentrations of ammonium and glucose in culture medium, cell dry weight (CDW), and lipid accumulation of Mc-1552 and Mc-D6E during growth were determined and shown in Fig. 2. In general, the recombinant strain showed a similar and typical growth profile as the control strain. Ammonium was used up at approx. 24 h by Mc-D6E but it was utilized more rapidly by the control strain Mc-1552 and glucose remained in excess during the entire bioprocess (Fig. 2a, b). CDW increased rapidly after 9 h of cultivation, and then slowed down after nitrogen exhaustion (Fig. 2c). Immediately after nitrogen depletion from the growth medium, the fungi started to accumulate lipids; from 24 h, the total fatty acids (TFAs) content increased rapidly, reached at its peak at 72 h and then slowed down (Fig. 2d).

### DGLA accumulation in *D6E (GLELO)* overexpressing strains

The fatty acid composition in *D6E (GLELO)* overexpressing strains were presented in Table 1 and cell dry weight (CDW), total fatty acids (TFAs) and DGLA yields were mentioned in Table 2. In the transformants, DGLA production started at 24 h, and at 48 h its content reached at almost 5%. The highest percentage (5.72%) of DGLA was measured at 60 h (Table 1). However the yield was maximum at 72 h which was 74.61 mg/l as it is corresponding to the TFAs content (Table 2). In these recombinant fungi there were little changes in other fatty acid contents compared with the control strain (Table 1).

**Table 1:** The fatty acid composition in *D6E (GLELO)* overexpressing strains

Time	Fatty acid composition (relative %, w/w)					
Hour	C(16:0)	C(18:0)	C(18:1) OA %	C(18:2) LA %	C(18:3) GLA %	C(20:3) DGLA %
*Mc-1552*						
12 h	–	–	32.38 ± 1.33	30.87 ± 0.96	36.75 ± 0.88	–
24 h	29.10 ± 0.75	7.84 ± 0.58	20.43 ± 0.44	16.50 ± 0.67	26.13 ± 0.37	–
36 h	29.91 ± 0.52	7.60 ± 0.27	22.57 ± 0.11	14.39 ± 0.38	25.53 ± 0.23	–
48 h	22.23 ± 0.64	4.32 ± 0.44	26.68 ± 0.32	16.56 ± 0.25	30.21 ± 0.10	–
60 h	24.72 ± 0.07	3.70 ± 0.15	25.85 ± 0.08	15.78 ± 0.05	29.95 ± 0.05	–
72 h	24.92 ± 0.25	2.71 ± 0.32	26.09 ± 0.22	15.68 ± 0.30	30.60 ± 0.20	–
*Mc-D6E*						
12 h	33.08 ± 1.78	7.56 ± 2.03	16.70 ± 1.1	18.44 ± 1.23	24.19 ± 0.86	–
24 h	32.45 ± 0.52	6.59 ± 0.88	18.28 ± 0.93	17.57 ± 1.10	23.37 ± 0.68	1.72 ± 0.88
36 h	25.95 ± 1.98	3.89 ± 0.91	26.92 ± 1.43	15.35 ± 0.93	24.20 ± 1.32	3.66 ± 1.52
48 h	26.87 ± 0.08	4.79 ± 0.26	21.16 ± 0.00	16.02 ± 0.33	26.19 ± 0.07	4.94 ± 0.07
60 h	24.99 ± 0.72	4.07 ± 0.32	24.60 ± 0.44	15.48 ± 0.50	26.39 ± 0.64	5.72 ± 0.77
72 h	27.28 ± 0.04	9.43 ± 0.61	21.48 ± 0.10	14.48 ± 0.67	21.59 ± 0.45	5.16 ± 0.13

The values represent the mean ± SD of three independent experiments

**Table 2:** The cell dry weight (CDW), total fatty acids (TFA) and DGLA yields in *D6E (GLELO)* overexpressing strains

Cultivation time (hour)	CDW (g/l)	TFA (g/l)	DGLA (mg/l)
12 h	4.15 ± 0.68	0.17 ± 0.55	–
24 h	6.20 ± 0.43	0.34 ± 0.67	5.81 ± 0.83
36 h	6.54 ± 1.27	0.69 ± 1.77	25.13 ± 1.68
48 h	7.80 ± 0.05	0.95 ± 0.04	46.81 ± 0.09
60 h	8.67 ± 0.89	1.20 ± 0.66	68.69 ± 0.59
72 h	9.18 ± 0.23	1.45 ± 0.33	74.61 ± 0.19

### Expression levels of *D6E (GLELO)* gene in the overexpressing strains

**Fig. 3:**
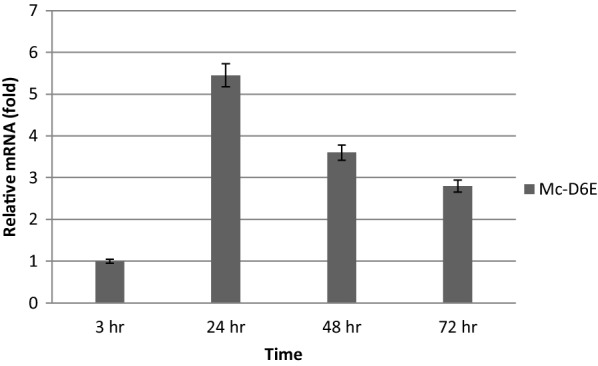
Determination of expression levels of *D6E (GLELO)* genes by RT-qPCR in the overexpressing strains

Real-time quantitative PCR were carried out to analyze the mRNA level of *D6E (GLELO)* in the selected overexpression strains at 3, 24, 48 and 72 h of growth in 2 l fermenter with K&R medium (Fig. 3). The mRNA expression level of Mc-D6E was considered as 1 at 3 h and by comparing with this value the expression level was increased respectively by 5.45, 3.6 and 2.8 fold at 24, 48 and 72 h in overexpressing strains. Although it increased quickly from 3 to 24 h but there was a decreasing trend with the incubation time after 24 h. The fact that D6E mRNA was maintained at elevated levels throughout the whole culture time confirmed that it was overexpressed in the recombinant strains.

## Discussion

Very-long-chain polyunsaturated fatty acid (VLCPUFA) production in transgenic organisms is a process which can further enhance the production of desired products [20]. The oleaginous fungus, *M. circinelloides*, is attracting considerable interest as it produces oil rich in Gamma linolenic acid. On the other hand *M. alpina* produce both ARA and EPA. But in all other aspects, both fungi share similar properties and phenotype (filamentous) [3]. Fatty acid elongase, *GLELO* of *M. alpina* ATCC 32221, is reported to convert 18-carbon PUFAs to the corresponding 20-carbon ones [21]. Conversion of GLA to DGLA is one step process catalyzed by Delta 6-elongase enzyme [3] encoded by *D6E* gene which we have cloned from *M. alpina* and expressed in *M. circinelloides*. Previously Delta5-Desaturase-defective mutant of *M. alpina* 1S-4 exhibited increased DGLA accumulation with low concentrations of ARA but because of their similar properties separation of DGLA from ARA is difficult during PUFA purification [22]. Previous work on metabolic engineering of long chain-polyunsaturated fatty acid biosynthetic pathway was done in *Saccharomyces cerevisiae* and *Aspergillus oryzae* for DGLA production but its concentration was low (less than 3%) [1, 11]. Our recombinant *M. circinelloides* CBS277.49 showed superior advantages as it produce higher DGLA (5.72%).

In most oleaginous microorganisms, nitrogen (N) deficiency is a common strategy to trigger lipid accumulation. The amount of lipid increases under N starvation and lipid accumulation is often investigated by comparing the N rich phase to N deficiency phase in the entire bioprocess [23–25]. The maximum concentration of TFAs is 15% in CBS 277.49 [7]. Similar trends and results were observed in our experiments. Manipulation of the expression of *D6E* genes had no significant effect on the growth and total lipid content (Fig. 2).

RT-qPCR results revealed that *D6E* mRNA were maintained at elevated levels throughout the whole culture time of Mc-D6E and fatty acid analysis showed the presence of DGLA. These results confirmed that *D6E* was overexpressed in the recombinant strains and the mRNA of the genes encoding *D6E* was increased upon N deficiency. In *M. circinelloides* the major fatty acids are 16:0, 18:0, 18:1, 18:2 (LA) and 18:3 (GLA) [7, 8]. After overexpressing *D6E* gene the same fatty acids were found as the major lipids and its expression did not affect the major lipid profile of this organism.

Researches has confirmed that deficiency of DGLA has been associated with various physiologic and pathophysiologic conditions including aging, diabetes, alcoholism, atopic dermatitis, premenstrual syndrome, rheumatoid arthritis, cancer and cardiovascular disease [26]. But the lack of sources for large scale production has prevented its scientific research and, as a result, its neutraceutical or pharmaceutical use. DGLA normally occurs only as an intermediate in the biosynthesis of ARA in higher plants or fungi and algae; it is not significantly accumulated in any organism. Instead, GLA-rich oils from several plant species are utilized as a DGLA precursor. Under certain conditions (e.g., calcium deficiency) the conversion of GLA to DGLA in the body is significantly diminished and GLA cannot replace DGLA. So the administration of DGLA is assumed to be more effective than the administration of GLA [27]. In our recombinant *M. circinelloides* DGLA is the only 20 carbon fatty acid that can be purified easily from other PUFA. Thus, the Mc-D6E may represent a promising source of DGLA for further up-scaling and optimization of its industrial cultivation.

In summary, we successfully constructed a DGLA-producer *M. circinelloides* CBS277.49 by *D6E (GLELO)* gene overexpression. To the best of our knowledge, this is the first study to report the construction of DGLA-producing transformant using the successful gene cloning and recombination system in *Mucor*. The DGLA producer may have applications in industrial DGLA production. This work established a new scope for further research for improved production of DGLA in *M. circinelloides,* specifically in its high lipid-producing strain, WJ11.

## Methods

### Strains, plasmids and culture conditions

*Mortierella alpina* ATCC 32222 was used as the source of *D6E (GLELO)* gene. The uracil auxotroph strain, pleu-MU402 of *M. circinelloides* CB277.49 [28] was used as the recipient strains in transformation experiments to overexpress the elongase gene. Escherichia coli strain Top 10 was used for all cloning experiments and grown in lysogeny broth at 37 °C. Plasmids pMAT1552 [29] were used as the cloning and expression vectors. *Mortierella alpina* ATCC 32222 was grown in 1 l flask containing Potato Dextrose Water (PDW) for 72 h for mycelia collection. The recombinant strains Mc-D6E (*D6E* overexpresssion strains) and Mc-1552 (strains carrying the vector pMAT1552) as the control were initially cultivated in 1 l flasks having 150 ml K & R medium containing 30 g/1 glucose, 3.3 g/1 diammonium tartrate, 7.0 g/1 KH_2_P0_4_, 2.0 g/1 Na_2_HP0_4_, 1.5 g/1 MgS0_4_∙7H_2_0, 1.5 g/1 yeast extract, 0.1 g/l CaC1_2_∙2H_2_0, 8 mg/l FeC1_3_∙6H_2_0, 1 mg/l ZnS0_4_. 7H_2_0, 0.1 mg/l CuSO_4_∙5H_2_0, 0.1 mg/l Co(NO_3_)_2_∙6H_2_0 and 0.1 mg/l MnS0_4_∙5H_2_0, pH 6.0 [30] for 24 h at 30 °C with shaking at 150 rpm and then inoculated at 10% (v/v) into a 2 l fermenter (BioFlo/CelliGen115, New Brunswick Scientific, Edison, NJ, USA) containing 1.5 l K & R medium modified to contain 80 g glucose/l. Fermenters were held at 28 °C, stirred at 700 rpm, with aeration at 0.5 vvm. The pH was maintained at 6.0 by automatic addition of 2 M NaOH for CBS277.49.

### Plasmid construction and the cloning strategy

**Fig. 4:**
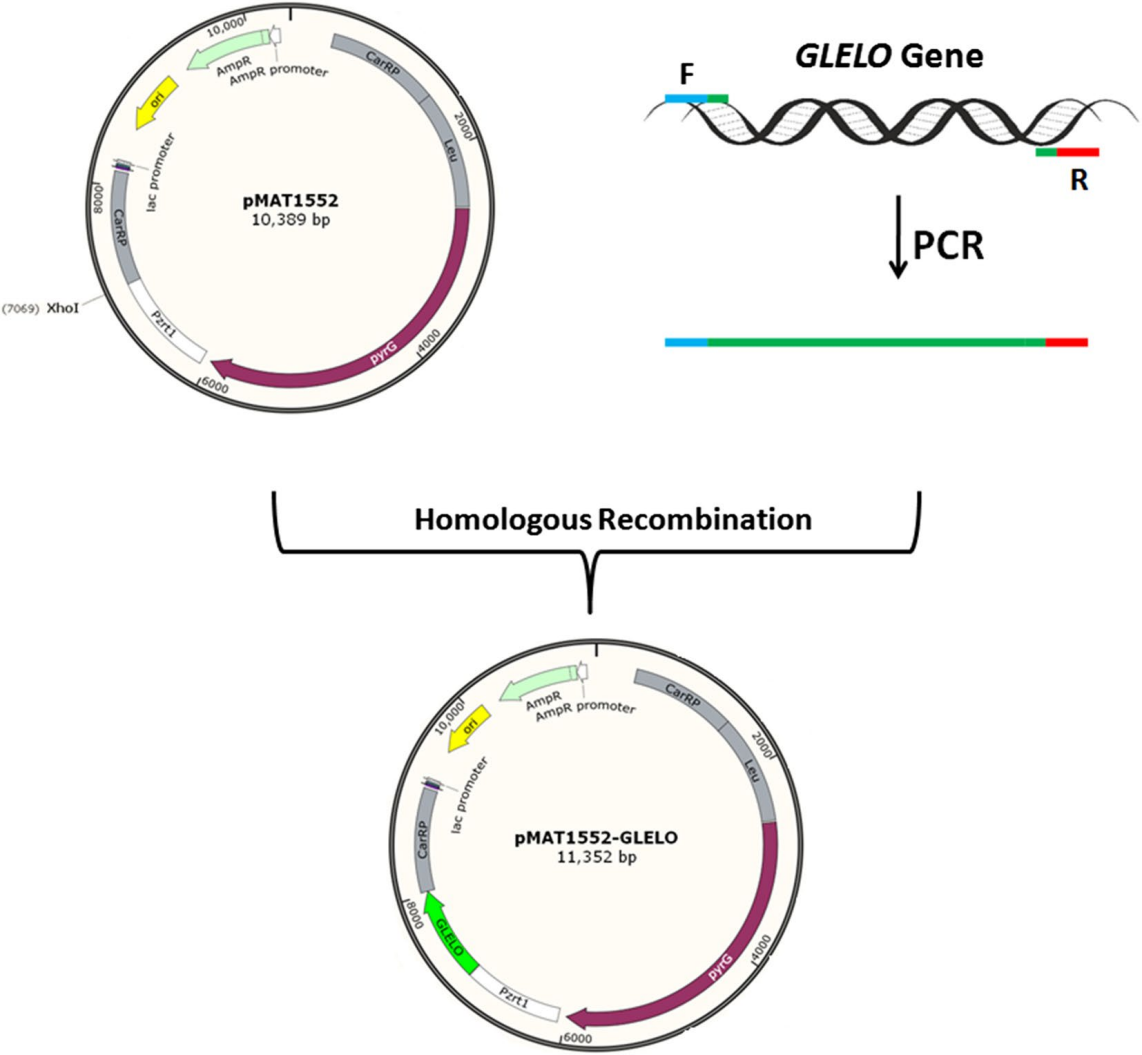
Structure of plasmids pMAT1552 and pMAT1552-GLELO are shown. The *D6E* (*GLELO)* gene was isolated by PCR amplification with appropriate primers. The PCR fragment was ligated into XhoI restriction site to generate plasmid as pMAT1552-GLELO

Plasmid, pMAT1552, that contains the *M. circinelloides pyrG* gene surrounded by up- and down-stream 1 kb of *carB*-*carRP* sequences, was used for construction of *D6E (GLELO)* overexpressing plasmids for pleu-MU402. The *pyrG* encodes orotidine 5’-phosphate decarboxylase which produce uridine as a selectable marker and flanking sequences corresponding to regions surrounding the carotenogenic *carRP*–*carRP* gene allows its chromosomal integration by homologous recombination [8, 29]. The *D6E* (*GLELO)* gene was isolated by PCR amplification from the genome of *Mortierella alpina* ATCC 32222 with corresponding primers GLELO-F/R (Additional file 1: Table S1). The primers contain 30 bp homologous sequences of both sides of XhoI restriction site in pMAT1552. After digestion the plasmid with XhoI, The PCR fragment was ligated into this site to generate plasmid as pMAT1552-GLELO for mutant pleu-MU402 (One step cloning kit, Takara). During experiment of gene cloning the ligation mixture was used to transform into chemically competent *E. coli* Top 10 cells. The plasmids isolated from these transformants were verified by doing PCR and the gene sequences were confirmed by DNA sequencing. The cloning strategy with the complete map of pMAT1552 and pMAT1552-GLELO were presented in Fig. 4.

### Transformation method

The electroporation-mediated transformation was done according to the procedure described by Gutiérrez et al. [31] with little modification. Fresh Spores (no more than 1 week old) from uracil auxotroph strain, pleu-MU402 of *M. circinelloides* CB277.49 were collected and 12.5 × 10^7^ spores were inoculated into 25 ml (250 ml flask) of the medium YPG plus uridine. After maintaining at 4 °C overnight without shaking, the solution was incubated at 28 °C and 200 rpm in a rotary shaker for spore germination (3.5 to 4 h). Germinated spores were recovered by centrifugation at 1100 rpm for 5 min at room temperature and washed with 5 ml of PS (0.01 M sodium phosphate buffer pH 6.5; 0.5 M sorbitol) by centrifugation at 1750 rpm for 5 min at room temperature.

To digest the cell wall, germinated spores were resuspended in 5 ml of PS treated with 0.3 μl chitosanase RD (C0794, Sigma-Aldrich) and 1 mg/ml lysing enzymes (L-1412; Sigma-Aldrich) at 30 °C with gentle shaking (60 rpm) for 90 min. From this step the cells were handled with extreme care. Cell wall removal was monitored by following the loss of cell wall-associated refringence in protoplasts using a phase-contrast microscope. To stop the cell wall digestion, 10 ml of ice cold 0.5 M sorbitol was added. The protoplasts were recovered by centrifugation at 4 °C and 1100 rpm for 5 min, resuspended in 5 ml of 0.5 M cold sorbitol and centrifuged again in the same conditions. Finally a volume of 800 μl of 0.5 M sorbitol was added to these protoplasts and these were used for electroporation and allows for eight different transformation experiments.

For electroporation, 1 μg of circular plasmid in a volume of 10 μl were added to 100 μl of protoplasts and the mixture was transferred to a 2 mm electroporation cuvette. An electrical pulse was applied to these cuvettes individually using the following conditions: field strength of 0.8 kV, capacitance of 25 μF, and constant resistance of 400 Ω. After applying an electrical pulse, 1 ml of cold YPG medium containing 0.5 M sorbitol was added to the electroporation cuvette immediately and the solution was transferred to a 1.5 ml eppendorf tube. After incubation for 1 h at 26 °C and 100 rpm, protoplasts were recovered by centrifugation at 1100 rpm for 5 min, resuspended in 500 μl of YNB containing 0.5 M sorbitol, and spread on selective medium plates (MMC plus 0.5 M sorbitol) and incubated in the dark at 28 °C for 3 to 4 days.

### Determination of glucose and nitrogen in culture medium

Glucose concentration in the culture was measured using a glucose oxidase Perid-test kit according to the manufacturer’s instructions (Shanghai Rongsheng Biotech Co., Ltd.). Ammonium was assayed by the indophenol method [32].

### Determination of cell dry weight, total fatty acid and fatty acid analysis

Biomass was harvested on a weighed filter paper by filtration through a Buchner funnel under reduced pressure and washed three times with distilled water, frozen overnight at − 80 °C and then freeze-dried. The weight of the biomass was determined gravimetrically. Biomass was collected by filtration and dried by lyophilize. The weight of empty fatty acid extraction tube was taken. 20 mg dry weight was taken for cell fatty acids extraction. The total fatty acids were extracted with chloroform/methanol (2:1, v/v) and weight of tube with fatty acid was taken. Total fatty acid was determined using following equation:

$$ Total\,FA = \frac{{{\text{T}}1 - {\text{T}}0}}{\text{Wm}} \times 100 $$

Here, T_1_ = Weight of tube with fatty acid, T_0_ = Weight of empty tube, Wm = Weight of mycelia.

For fatty acid analysis 10 mg of dry mycellia was taken. Fatty acid was extracted by same method and methylated with 10% (v/v) methanolic HCl at 60 °C for 3 h. Pentadecanoic acid (15:0) was added into the freeze-dried cells as an internal standard. The resultant fatty acid methyl esters were extracted with n-hexane and were analyzed by GC equipped with a 30 m × 0.32 mm DB-Waxetr column with 0.25 μm film thickness. The program was as follows: 120 °C for 3 min, ramp to 200 °C at 5 °C per min, ramp to 220 °C at 4 °C per min, hold 2 min.

### Gene expression and RT-qPCR analysis

For reverse transcription-quantitative PCR (RT-qPCR) analysis, strains were grown in a 2 l fermenter with K & R medium, and the mycelium was harvested at 24, 48, and 72 h. Total RNA of *M. circinelloides* was extracted with Trizol after grinding under liquid N_2_ and reverse-transcribed using the Prime ScriptRT reagent kit (Takara) according to the manufacturer’s instructions. RT-qPCR was performed using primers GLqPCR-F/R (Additional file 1: Table S1) on LightCycler 96 Instrument (Roche Diagnostics GmbH, Switzerland) with FastStart Universal SYBR Green Master (ROX) Supermix (Roche) according to the manufacturer’s instruction. The mRNA expression level was normalized to levels of 18S rRNA, and the results were expressed as relative expression levels. The data were quantified by the method of 2^−ΔΔCt^.

### Statistical analysis

A statistical analysis was carried out using SPSS 16.0 for Windows (SPSS Inc. Chicago, IL). The mean values and the standard error of the mean were calculated from the data obtained from three independent experiments. The differences between the means of the test were evaluated by Student’s *t* test, and *P *< 0.05 was considered as significantly different.

## Additional file

**Additional file 1: Table S1.** Primer sequences used in this study.

## Abbreviations

OA: oleic acid
LA: linoleic acid
GLA: gamma linolenic acid
DGLA: dihomo-gamma linolenic acid
ARA: arachidonic acid
EPA: eicosapentaenoic acid
DPA: docosapentaenoic acid
DHA: docosahexaenoic acid
GLELO: gamma linolenic acid elongase
D6E: delta-6 elongase
PUFA: polyunsaturated fatty acid
GC: gas chromatography
VLCPUFA: very-long-chain polyunsaturated fatty acid
CDW: cell dry weight
SAFA: saturated fatty acids
MUFA: mono-unsaturated fatty acids
EFAs: essential fatty acids
