# Supplementary material for: Construction of DGLA producing cell factory by genetic modification of Mucor circinelloides
Source: Microb Cell Fact. 2019 Apr 3;18:64. doi: 10.1186/s12934-019-1110-4 (PMC6448318; doi:10.1186/s12934-019-1110-4)
Supplement: Supplementary file 1 — Additional file 1: Table S1. Primer sequences used in this study. [file 12934_2019_1110_MOESM1_ESM.docx]

| **Primer** | **Sequence (5ʹ to 3ʹ )^a^** | **Restriction enzyme** | **Application** |
| --- | --- | --- | --- |
| GLELO-F | ACTTTTATATACAAAATAACTAAATCTCGAGATGGAGTCGATTGCGCCATT | XhoI | Gene cloning |
| GLELO-R | ACTAGTCGCAATTGCCGCGGCTCGAGTTACTGCAACTTCCTTGCCT | XhoI | Gene cloning |
|  |  |  |  |
| GLqPCR-F | CAAGAAGAACAACCGCCAGATCTCC |  | RT-PCR analysis |
| GLqPCR-R | CCGTACATGATCACATGGATGAACG |  | RT-PCR analysis |
|  |  |  |  |
| 1552-F | CCTCGGCGTCATGATGTTTTTGTGTACCT |  | Gene checking |
| 1552-R | GGGATGTCTGCTGCTACCATGTCTCAT |  | Gene checking |

^a^ Restriction enzyme sites are underlined.
